# Challenges on the epidemiological and economic burden of diabetes and hypertension in Mexico

**DOI:** 10.11606/S1518-8787.2018052000293

**Published:** 2018-02-07

**Authors:** Armando Arredondo, Emanuel Orozco, Jaqueline Alcalde-Rabanal, Juan Navarro, Alejandra Azar

**Affiliations:** I Instituto Nacional de Salud Pública. Cuernavaca, Morelos, Mexico; II Universidade Federal da Bahía. Instituto de Saúde Coletiva. Salvador, BA, Brasil

**Keywords:** Diabetes Mellitus, epidemiology, Hypertension, epidemiology, Health Services Needs and Demand, economics, Healthcare Financing, Diabetes Mellitus, epidemiología, Hipertensión, epidemiología, Necesidades y Demandas de Servicios de Salud, economía, Financiación de la Atención de la Salud

## Abstract

**OBJECTIVE:**

To analyze the epidemiological and economic burden of the health services demand due to diabetes and hypertension in Mexico.

**METHODS:**

Evaluation study based on a time series study that had as a universe of study the assured and uninsured population that demands health services from the three main institutions of the Health System in Mexico: The Health Department, the Mexican Institute of Social Security, and Institute of Services and Social Security for State Workers. The financing method was based on instrumentation and consensus techniques for medium case management. In order to estimate the epidemiological changes and financial requirements, a time series of observed cases for diabetes and hypertension 1994–2013 was integrated. Probabilistic models were developed based on the Box-Jenkins technique for the period of 2013–2018 with 95% confidence intervals and p < 0.05.

**RESULTS:**

Comparing results from 2013 *versus* 2018, in the five regions, different incremental trends of 14%–17% in epidemiological changes and 58%-66% in the economic burden for both diseases were observed.

**CONCLUSIONS:**

If the risk factors and the different models of care remained as they currently are in the three institutions analyzed, the financial consequences would be of greater impact for the Mexican Institute of Social Security, following in order of importance the Institute of Services and Social Security for State Workers and lastly the Health Department. The financial needs for both diseases will represent approximately 13%–15% of the total budget allocated to the uninsured population and 15%–17% for the population insured depending on the region.

## RESUMEN

**OBJETIVO:**

Analizar la carga epidemiológica y económica de la demanda de servicios de salud por diabetes e hipertensión en México.

**MÉTODOS:**

Investigación evaluativa basada en un estudio de series de tiempo que tomó como universo de estudio la población asegurada y no asegurada que demanda servicios de salud a las tres principales instituciones del Sistema de Salud en México: Secretaría de Salud, Instituto Mexicano del Seguro Social, e Instituto de Servicios y Seguridad Social para los Trabajadores del Estado. El método de costeo tomó como base las técnicas de instrumentación y de consenso por manejo de caso promedio. Para estimar los cambios epidemiológicos y requerimientos financieros, se integró una serie de tiempos de casos observados para diabetes e hipertensión 1994–2013. Se desarrollaron modelos probabilísticos basados en la técnica de Box-Jenkins para el periodo 2013–2018 con intervalos del 95% de confianza y p < 0.05.

**RESULTADOS:**

Comparando resultados de 2013 *versus* 2018, en las cinco regiones, se observaron diferentes tendencias incrementales de 14%–17% en cambios epidemiológicos y de 58%–66% en la carga económica para ambas enfermedades.

**CONCLUSIONES:**

Si los factores de riesgo y los diferentes modelos de atención permanecieran como están actualmente en las tres instituciones de análisis, las consecuencias financieras serían de mayor impacto para el Instituto Mexicano del Seguro Social, siguiendo en orden de importancia el Instituto de Servicios y Seguridad Social para los Trabajadores del Estado y finalmente para la Secretaría de Salud. Los requerimientos financieros para ambas enfermedades representarán aproximadamente del 13%–15% del presupuesto total asignado para población no asegurada y el 15%–17% para población asegurada dependiendo de la región.

## INTRODUCTION

Within the framework of effective universal coverage, the increase in the costs of health services for the treatment of noncommunicable diseases, the need to increase investment, the lack of financial protection of the health services user, and the urgency of changes in the methods of resources distribution in the health sector have aroused multiple concerns among decision makers^1^ ^,^ ^2^. The demand for health care does not diminish and the high cost of the different alternatives of action in this period of transitions in all areas imposes a heavy burden on the national and state budgets that governments are trying to reduce^3^.

Cardiovascular diseases in people with diabetes mellitus begin earlier, and present with atypical symptoms and signs^4^. The complications of chronic diseases such as diabetes and hypertension increase significantly. The investment of health systems to treat diabetes should include not only the management of the disease but also its complications. Diabetes is a worldwide emergency due to excessive spending that affects the sustainability of health systems. Complications of noncommunicable diseases are costly: diabetic nephropathy has been shown to be the most costly complication of diabetes in the Americas^4^.

In economic terms, the significance of the changes in the epidemiological and demographic profile is an increase in the care demand for costly diseases (treatment of chronic degenerative diseases and accidents). This will compete with the budget allocated for the treatment of infectious diseases still unresolved^5^ ^,^ ^6^. Thus, there will be a need to develop and apply analytical tools to reassess health priorities and establish strategic actions for the allocation, use and adequate organization of financial resources in the health sector^7–9^.

This article aimed to analyze the epidemiological and economic burden of the health services demand due to diabetes and hypertension in Mexico. We analyzed the epidemiological changes expected for the period of 2013–2018, as well as the financial requirements for the two study demands at the Mexican Social Security Institute (IMSS), the Health Department (SSA) and the Institute of Services and Social Security for State Workers (ISSSTE) at the level of each state health system.

## METHODS

Evaluation research based on a time series study in Mexico for the period of 2013–2018. The selected states were Jalisco, Hidalgo, Morelos, Sinaloa, and Yucatan. The states studied showed relative heterogeneity for the indicators of marginalization, public insurance, epidemiological delay and original population index, in the macroeconomic indicators of socioeconomic development^10^ ^,^ ^11^ (Table 1). The indexes of epidemiological delays and original population represented greater challenges in all cases since the highest index of marginalization coincided with the highest epidemiological delay, which is usually more urgent in the original population.

**Table 1:** Sociodemographic characteristics of selected states. Cuernavaca, 2016.

State	Total population	Marginalization Index	Public Insurance Index	Epidemiological Attainment Index	Indigenous Population Index
Sinaloa	2,966,321	Low	High	Low	Low
Morelos	1,903,811	Medium	Medium	Medium	Low
Hidalgo	2,858,359	High	Medium	Medium	Medium
Jalisco	7,844,830	Low	High	Low	Low
Yucatan	2,395,272	High	Medium	High	High

Sources: National Population Council – National Institute of Statistics and Geography, 2000–2015. Health Department, Statistical Yearbook on Health Damage, 1998–2015. Information for the Provision of Accounts, Mexico, 2006-2015. Population Census 2010–2015.

Study institutions included IMSS, SSA, and ISSSTE. The annual demand for health services for hypertension and diabetes was obtained from the number of cases in control for diabetes and hypertension reported by type of institution. This information was obtained from the statistics bulletin on health damages of the National Health System, 1994–2013^12^.

The direct costs of care were obtained from standard case management and adjusted by type of institution. The financing method was based on the methodology of related diagnostic groups (GRD [Group of Related Diagnostic] variant adjusted for Mexico)^13^ and based on the instrumentation technique. Once the work teams were integrated and the diseases to be covered (diabetes and hypertension) had been selected, we chose to work with the consensus technique to determine the mean case management. In order to do this, we discussed the pilot test, the collection instruments that, according to the natural history of the disease and the work experience of the multidisciplinary team, were redesigned to capture the different processes, procedures and resources (production functions and materials), as well as all ex-ante and ex-post activities of each production function to provide the service that each pathology requires. The participation of physicians was fundamental for determining the different medical care processes and for the fact that the quality of care offered at the hospital and at different levels of care was debated from the perspective of the provider.

The data collection instruments for each production function included different categories of material and cost analysis: infrastructure, human resources in medical hours, nurse hours and administrative hours, laboratory studies, complementary studies, average stay-day-bed, components of drug-curative therapy, intensive care components, contracted services, general services, other resources, processes and procedures, and observations. The materials and costs were concentrated in financing matrices by type of illness and type of institution.

This technique was used to establish consensus and standardize the methodology for obtaining production functions and materials. The professionals of the different areas (clinical, epidemiological and administrative) had an active and determinant participation in the management of the mean case by illness. Every member of the work team had at least five years of clinical-administrative practice experience.

To determine the financial requirements of expected cases, a time series 1994–2013 was carried out. Autoregression models were designed using the Stat Graphics software under the Box-Jenkins technique^14^ and with 95% confidence intervals and p < 0.05. The proposed method was justified because the case observations showed to be highly correlated. This is because we have available data recorded for diabetes and hypertension with the quality and quantity required to apply this technique. In addition, it is one of the most recommended methods for medium- and long-term projections in the analysis of chronic public health problems. The development of the models considered each of the following methodological steps and phases:

**Step 1: Identification**

- Identification of the tentative model of the time series for use in prediction.
- Review of information quality and number of observations for diabetes and hypertension.
- Self-correlation analysis of historical observations.

**Step 2: Estimate**

- Determination of parameter estimators, using the least squares criterion.
- Application of an iterative procedure to search for a function of the sum of squares, specifying previously the preliminary estimators of the unknown parameters.

**Step 3: Verification of diagnosis**

- Proof of suitability, after the models are adjusted to the data.
- Analysis of the difference between the observed and the expected in cases of diabetes and hypertension.
- Application of the Box-Pierce chi-squared.

**Step 4: Prediction**

- Selection and design of the definitive model.
- Data processing.
- Prediction of definitive expected values for the time series, both in diabetes and in hypertension

The selection of models and prediction equations were designed with cases observed up to 2012. Once a results report was prepared, it was ratified to update the projections with the cases observed until December 2012, projecting for the period of 2013–2018. These results were used to analyze the financial implications of epidemiological changes for hypertension and diabetes in each institution and the state health system under study.

To estimate the financial consequences of changes in demands by type of institution and disease, an inflation index projected for 2013–2018 was applied, based on the Bank of Mexico consumer price index^15^. The results were presented in National Currency and US dollars at the exchange rate in June 2015 and with an exchange rate of US $1 = Mex $14.35.

## RESULTS

The results for the case of diabetes and hypertension were similar in the model development except in the number of cases per type of disease, as will be verified later. The historical data were the number of monthly cases of diabetes in the period from 1996 to 2012. Monthly case numbers were denoted by Y1, Y2,..., Y84. The number of cases followed a pattern with a strong upward trend until the year 2000, declining sharply in 2001 and increasing again in 2002, with irregular peaks until 2012. The slope coefficient in trend analysis was positive and significantly different from zero (t = 6.76). The time series was described by the trend factor and the number of cases of diabetes tended to increase.

On the autocorrelation function of the original series, the series was not stationary, because the autocorrelation function slowly declined. After applying the transformation of a non-seasonal difference, the time series seems to be stationary and can be explained by the transformation Zt = Yt - Yt-1 which means a non-seasonal difference.

It seems that the autocorrelation function intersects the line representing the boundary of non-zero statistical significance after lag 1 and that the partial autocorrelation function dies quickly. This indicates the need to include an average order operator of order 1. Another interpretation of both functions is that the autocorrelation function dies quickly and that the partial autocorrelation function is cut in lag 1. Thus, the time series could be explained by an autoregressive operator of order 1.

As a result of the analysis of the functions of autocorrelation and partial autocorrelation, the following models were proposed:

Model 1 – Medium movement operator of order 1:

Z_τ_ = δ = ε_τ_ − θ_1_ ε_τ_-1

Model 2 – Autoregressive operator of order 1:

Z_τ_ = δ + f_1_ Z_τ_-1

The results of the two models were presented in Table 1. We included the parameter estimators in each model, the t-statistic, the Box-Pierce chi-squared statistic in the first 20 residuals, and we indicated the lags in which the residuals had significantly different non-zero correlations. The standard separation value (s) was presented for each model. The two models fit the data well and did not have self-correlations of residuals significantly different from zero. Model 2 was selected as the definitive model because it was the one with the lowest standard error (Table 2).

**Table 2:** Results of statistical proofs of estimative models of diabetes and hypertension cases. Cuernavaca, 2016.

Variable	Model 1	Model 2
Number of non-stationary differences	1	1
Number of stationary differences	0	0
Number of parameters	3	2
Estimator A	0.7689 (4.01)	0.2579 (2.43)
Estimator B	0.5536 (2.23)	-0.2723 (-2.54)
Estimator C	0.9123	0.8599
Box-Pierce chi-squared	14.67	22.25
Standard deviation	26.9	21.22
Correlations other than zero	0	0

With the results of this adjustment, it was demonstrated that the autoregressive operator of order 1 adequately described the cases of diabetes mellitus, since the difference between observed and estimated was small. As the slope value of the series resulted significantly different from zero (t = 6.76), a constant term was used when adjusting the model. This coincides with the judgments of the health planners, who consider that chronic illnesses tend to increase, that is, that the rate of increase tendency will continue in the future in the short, medium and long-term. For this reason, both models included this term. The appropriate model was Model 2, with the prediction equation.

γ+_τ_ = δ + g_τ-13_ + b_1,12_ ε_τ-13_ − q_1_ ε_τ-1_ + q_1_q_1,12_ε_τ-13_ + ε_τ_

Where:

γ+_τ_ = The annual average of expected cases

δ = Partial autocorrelation residual factor

g_τ-13_ = The historical value of the series of years prior to prediction

b_1,12_ ε_τ-13_ = Partial autocorrelation coefficient of the time series

q_1_ ε_τ-1_ = Number of cases per type of estimator (A, B or C)

q_1_q_1,12_ε_τ-13_ = Average value of the series by total autocorrelation

ε_τ_ = The critical value of the standard deviation

In all the states, we observed epidemiological trends of observed and expected cases for constant increasing diabetes and hypertension, with irregular peaks before 2012, especially in Jalisco, where the greatest epidemiological burden was observed in absolute terms (Table 3). The difference in diabetes, in the case of hypertension, the first place in terms of the number of cases, were shared by Jalisco and Sinaloa. Morelos, Yucatan, and Hidalgo presented constant trends, slightly increasing and without irregular peaks. The irregular trends were found more in the case of Jalisco and Sinaloa for the first part of the period.

**Table 3:** Number of cases expected by type of disease at the state level (cases in absolute value). Cuernavaca, 2013–2018.

Disease/State	Number of cases per year						p

	2013	2014	2015	2016	2017	2018	
Diabetes							

Sinaloa	16,126	15,524	17,457	16,677	18,270	18,496	< 0.01
95%CI*	15,926–16,326	15,114–15,934	16,937–17,977	15,997–17,357	17,530–19,010	17,646–19,346	
Morelos	10,618	10,803	11,660	11,982	12,681	12,975	< 0.01
95%CI*	10,418–10,818	10,393–11,213	11,140–12,180	11,302–12,662	11,941–13,421	12,125–13,825	
Hidalgo	9,232	9,605	9,864	10,355	10,686	11,124	< 0.01
95%CI*	9,032–9,432	9,195–10,015	9,344–10,384	9,675–11,035	9,946–11,426	10,274–11,974	
Jalisco	19,154	20,794	21,454	21,996	22,352	22,666	< 0.01
95%CI*	18,954–19,354	20,384–21,204	20,934–21,974	21,316–22,676	21,612–23,092	21,816–23,516	
Yucatan	6,174	6,409	6,382	6,700	6,980	7,172	< 0.01
95%CI*	5,974–6,374	5,999–6,819	5,862–6,902	6,020–7,380	6,240–7,720	6,322–8,022	

Hypertension							

Sinaloa	28,017	29,673	31,406	33,018	34,303	35,424	< 0.01
95%CI*	27,817–28,217	29,263–30,083	30,886–31,926	32,338–33,698	33,563–35,043	34,574–33,274	
Morelos	15,353	15,645	16,010	16,196	17,392	17,619	< 0.01
95%CI*	15,153–15,553	15,235–16,055	15,490–16,530	15,516–16,876	16,652–18,132	16,769–18,469	
Hidalgo	9,992	10,246	10,422	11,107	11,441	11,748	< 0.01
95%CI*	9,792–10,192	9,836–10,656	9,902–10,942	10,427–11,787	10,701–12,181	10,898–12,598	
Jalisco	26,982	30,851	31,169	31,658	32,116	32,477	< 0.01
95%CI*	26,782–27,182	30,441–31,261	30,649–31,689	30,978–32,338	31,376–32,856	31,627–33,327	
Yucatan	8,953	9,222	9,465	9,620	9,785	10,022	< 0.01
95%CI*	8,753–9,153	8,812–9,632	8,945–9,985	8,940–10,300	9,045–10,525	9,172–10,872	

* 95% confidence intervals with Box-Pierce statistical test.

Constant growing trends in financial requirements were observed in all states. The increases occurred in a space between 8% and 40% depending on the year of analysis and the state. The state that presented the greatest financial requirements was Jalisco, followed by Sinaloa, Morelos, Hidalgo and lastly Yucatan, with an economic burden almost half of the penultimate state (Hidalgo). Comparing the state of greater economic burden (Jalisco) with the state of lower economic burden (Yucatan), the difference was more or less five to one.

The largest economic burden was presented by the IMSS, followed by the SSA and lastly the ISSSTE. The results detailing the costs and financial consequences attributable to hypertension appear in the attachment tables. It is important to note that the three institutions presented constant increasing trends for each of the years of the estimation period.

Differently from the economic burden of diabetes, hypertension presented the highest economic burden for the case in Sinaloa followed by Jalisco, Morelos, Hidalgo, and Yucatan in descending order. Comparing the spaces of economic impact by state, with Sinaloa as high impact and Yucatán as sub impact, the difference was more or less three to one. The trends of increases were similar to those of Diabetes in a space between 8.0% and 40.0% depending on the period of analysis.

**Figure 1:**
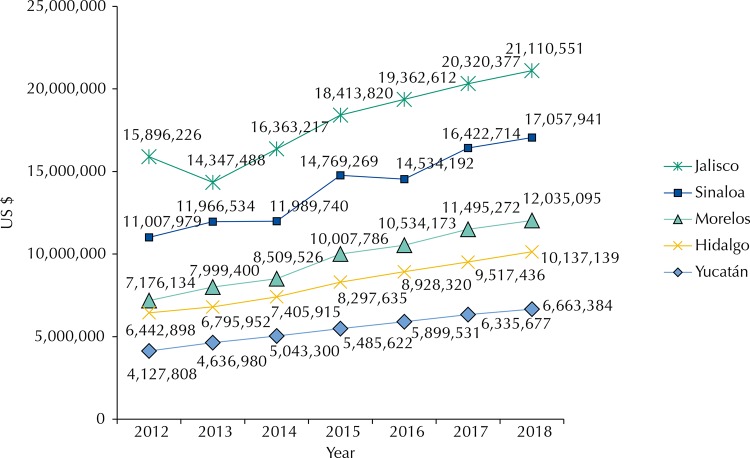
Total costs of diabetes in different states (in US$). Cuernavaca, 2012–2018.

**Figure 2:**
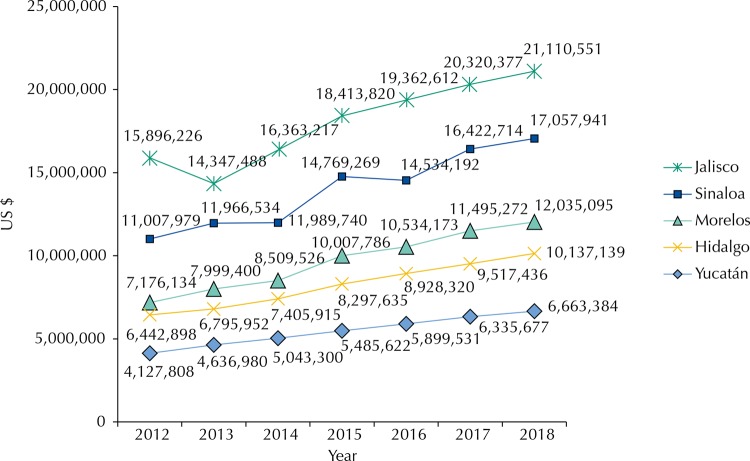
Total costs of hypertension in different states (in US$). Cuernavaca, 2012–2018.

**Figure 3:**
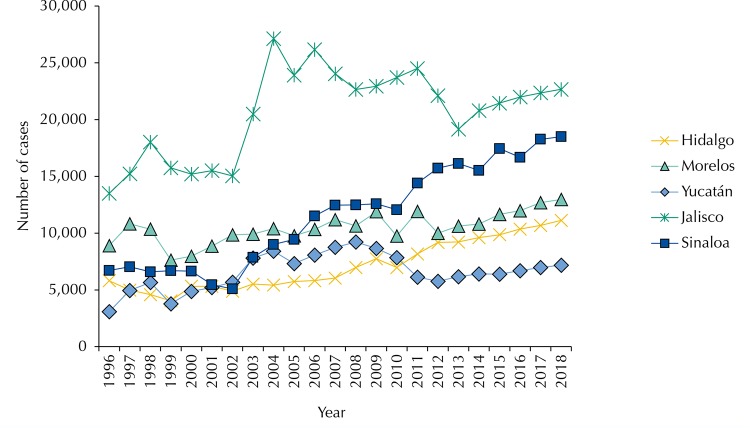
Trends in observed cases 1996–2012 and expected cases 2013–2018 for diabetes in the states under study. Cuernavaca, 2016.

**Figure 4:**
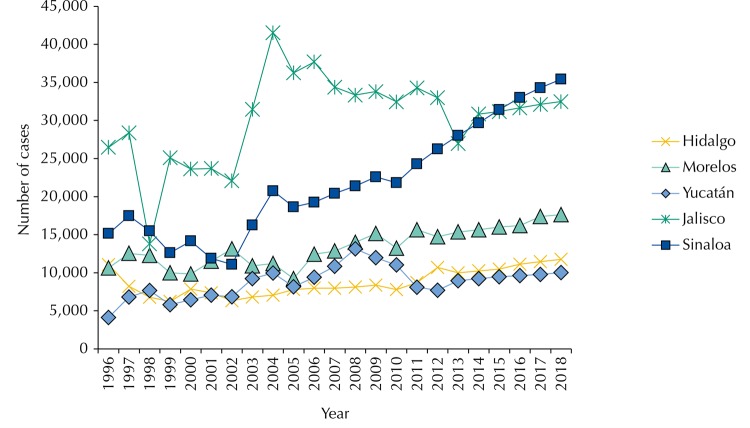
Trends in observed cases 1996–2012 and expected cases 2013–2018 for hypertension in the states under study. Cuernavaca, 2016.

Economic and epidemiological trends can be graphically observed in the period between 2012-2018 (Figures 1–4). For the diabetes case, the difference between the five states was evident, as well as the absence of irregular periods without overlap between the states. The order of importance was: Jalisco, Sinaloa, Morelos, Hidalgo, and Yucatan.

Exchange rate of 14.35 MEX $ per 1 US $ (Reference period June 2015).

Exchange rate of 14.35 MEX $ per 1 US $ (Reference period June 2015).

We noted the difference in the trend of combination of epidemiological and economic burden for diabetes mentioned at the beginning against the trend in prevalence rates. In fact, while in the combination of epidemiological trends and economic burden the first place was occupied by Jalisco, followed by Sinaloa, Morelos, Hidalgo, and Yucatan in decreasing order, in the case of prevalence, the first place was occupied by Yucatan, followed by Hidalgo, Sinaloa, Morelos and Jalisco. The trend in the states was totally reversed so that Yucatan ranked first and Jalisco last in order of importance in the impact.

For the hypertension case, while in the combination of the epidemiological and economic burden the greatest impact was for Sinaloa and Jalisco, followed by Yucatan, Hidalgo, and Morelos in decreasing order, in the case of an impact by prevalence there are similarities in trends. In fact, the highest prevalence was for Sinaloa followed by Hidalgo, Jalisco, Yucatan and Morelos in descending order. In both analyzes, the major and minor impact trends were for Sinaloa and Morelos, respectively.

## DISCUSSION

The trends of our findings in terms of epidemiological trends and economic impact coincide in important ways with other studies or similar analyzes^16–20^. Their analyzes coincide in the need to incorporate the economic evaluation within the health sector and to organize with greater efficiency the production of services prioritizing according to the real needs of the population that will make use of it. The trends of constant increases are justified by a number of reasons: the diversification of the demand for pressures in expected epidemiological exchanges, the scarcity of financial resources, and the varied and endless health needs that will be generated with the important changes in the epidemiological profile for the coming years in the majority of developing countries, especially in Latin America^21^ ^,^ ^22^.

The change in epidemiological profiles and the consequent increase in the demand for resources to treat these chronic diseases strongly suggest the need to adjust the care model so that cases are increasingly treated on an outpatient basis. The objective is to reduce the emergence of complications that require hospital management. An essential feature of the adjusted model would be to increase the value of outpatient care for the patient. This objective is in clear relation with the economic rationality of efficiency, just as it has resulted in similar studies of health costs for chronic diseases^17^ ^,^ ^23^.

Insurance systems are financed with public resources from taxes. Given the global economic crisis and its effects on health^24^, health budgets, particularly for Mexico and most Latin American countries, have declined significantly in recent years^25^. More than ever, efficient institutions are needed to allocate and manage resources. Likewise, it is required that the efficient use of resources guarantee the financial feasibility of strategies that minimize the costs of care linked to chronic diseases, that is, to direct resources to basically preventive and health promotion actions. In this sense, diabetes and hypertension need the implementation of care models oriented to the empowerment and active participation of users so that self-care favors the identification of complications in advance. These models that involve the patient and their family in the health care are highly effective because they can report in advance the emergence of a complication. Consequently, complications can be greatly reduced. This will have favorable consequences for the reduction or greater control of the costs of care related to these ailments and their complications. It is necessary to strengthen the first level of care to promote and strengthen a model focused on prevention and health promotion.

The results of our study show the relevance of incorporating epidemiological and economic aspects into the clinical perspective, periodically, the integral proposal for the analysis and evaluation of the performance of the health system in the context of sector reforms. The development of evaluation research studies that integrate an economic valuation with clinical and epidemiological valuations becomes relevant based on empirical expectations. As health reform projects proceed, the cost of lending services solely to the demand for hospital cases of chronic-degenerative diseases will be higher relative to the cost of providing service to the demand for outpatient and hospital cases of infectious diseases. This implies that the further advancement of the epidemiological transition will have greater financial consequences in the production of medical care services for future demands on diseases such as diabetes and hypertension.

The changes observed and expected in the epidemiological profile of chronic diseases in relation to infectious diseases will lead to a financial competence in the use of resources. The allocation of financial resources to produce services aimed at chronic diseases will be affected by the production of services for infectious diseases. Internal competence in the use and allocation of economic resources requires the approximate knowledge of the financial requirements to produce the services that will be demanded in the short and medium term. In this sense, the production and financing of health services will require incorporating clinical, epidemiological and economic indicators integrating these indicators under the criterion of efficiency depending on the context of each state’s health system.

The results allow us to make significant changes in the allocation of resources and adjustments to the model of care of the state’s health systems in function of the variables of epidemiological backwardness, relative weight of the indigenous communities, incorporation of cultural aspects in proposals of prevention and promotion models taking in consideration habits and customs in each region. This differential analysis will have important effects on the phenomenon of internal competence in the use and allocation of resources in different scenarios. For example, in Jalisco and Sinaloa, higher increasing trends of diabetes and hypertension were observed than in the rest of the states included in the study. This leads us to infer unequal growth trends in diabetes and hypertension in the states and that supposes three scenarios: states with high growth (Jalisco and Sinaloa), states with medium growth (Yucatan) and states with low growth (Hidalgo and Morelos). These scenarios will require research to explain the factors that influence the lower or higher growth of chronic diseases and support to implement integrated strategies in a care model that can curb the growth of this type of ailments.

The main limitation in time series studies refers to the availability of quality and quantity data in the number of observations. However, there was an adequate number of observations in this study to be able to make the estimates with a high degree of reliability. As we move to the last year of the period, both the epidemiological and economic burdens may have less precision and certainty; so we recommend a monitoring to update the time series and their respective epidemiological and economic burden at least every two years. Another limitation is that the estimated economic burden may vary as there are changes in medical technology and drugs to manage both diseases, possible changes to the protocol or possible changes to health programs, or by inflationary changes in the price index of materials. There is hope that these changes will not happen in five-year periods, and bi-annual monitoring could control these constraints.

In terms of relative frequencies, at national, state/provincial or institutional levels (institutions for insured persons and institutions for public assistance), the analysis of expected levels and epidemiological and economic trends can be replicated. The same trend of relative frequencies can be applied to the analysis of other chronic diseases of any country with epidemiological changes similar to Mexico since the diseases of the study were selected under the criterion of idealizers of the epidemiological transition. It can also be applied to other levels of intervention, diseases or other institutions of the health sector as the relative frequencies that correspond to each production function on the cost of handling interventions.

The changes in the epidemiological profile of Latin American countries raise doubts that one of the biggest challenges is the economic one. Both the internal competence and the patterns of resource allocation will be directly affected by the expected epidemiological changes. The costs of providing services aimed at new and more demanding chronic diseases, as well as competing with the classic demands of infectious diseases, require important changes in the patterns of resource allocation. The costs and financial consequences of the change in the epidemiological profile are analytical elements that should be taken into account in the planning of current and future health plans or programs at regional, national and state/state level, even if health sector spending is increased, especially when talking about the restructuring of public spending that raised doubts for the next years in each national and/or state health system.
